# Estradiol promotes the development of a fibrotic phenotype and is increased in the serum of patients with systemic sclerosis

**DOI:** 10.1186/ar4140

**Published:** 2013-01-10

**Authors:** Keiko Aida-Yasuoka, Christine Peoples, Hidekata Yasuoka, Pamela Hershberger, Katelynn Thiel, Jane A Cauley, Thomas A Medsger, Carol A Feghali-Bostwick

**Affiliations:** 1 Division of Pulmonary, Allergy, and Critical Care Medicine, Department of Medicine, University of Pittsburgh School of Medicine, 3459 Fifth Avenue, 628 NW MUH, Pittsburgh, PA 14213, USA; 2 Division of Rheumatology and Clinical Immunology, Department of Medicine, University of Pittsburgh School of Medicine, BST South 7th floor, Pittsburgh, PA 15261, USA; 3 Department of Pharmacology and Therapeutics, Roswell Park Cancer Institute, Elm & Carlton Streets, Buffalo, NY 14263, USA; 4 Department of Epidemiology, University of Pittsburgh Graduate School of Public Health, A510 Crabtree Hall, Pittsburgh, PA 15261, USA

## Abstract

**Introduction:**

Systemic sclerosis (SSc) is more prevalent in women. Our goal is to determine the effects of 17β-estradiol (E2) on the development of fibrosis and to compare circulating levels of estrogens in SSc patients and healthy controls.

**Methods:**

Using primary human dermal fibroblasts, we evaluated the effect of E2 on fibronectin (FN) expression with and without the estrogen receptor (ER) antagonist ICI 182,780, inhibitors of signaling, propyl-pyrazole-triol, an ERα specific ligand, and genistein, an ERβ selective ligand, to identify the signaling pathways mediating E2's effect. We confirmed the fibrotic effect of E2 in human skin using an *ex vivo *organ culture model. Lastly, we measured levels of E2 and estrone in serum samples from SSc patients with diffuse cutaneous involvement and healthy controls using mass spectrometry.

**Results:**

E2 increased expression of FN in dermal fibroblasts. ICI 182,780, inositol-1,4,5-triphosphate inhibitor, and p38 mitogen-activated protein kinase inhibitor blocked the effects of E2 on FN. Propyl-pyrazole-triol, but not genistein, significantly increased FN expression. *Ex vivo*, E2 induced fibrosis of human skin. The effects of E2 were abrogated by ICI 182,780. Circulating levels of E2 and estrone were significantly increased in sera of patients with diffuse cutaneous SSc.

**Conclusion:**

Our findings implicate estrogens in the fibrotic process and may explain the preponderance of SSc in women. ICI 182,780 or other ER signaling antagonists may be effective agents for the treatment of fibrosis.

## Introduction

Systemic sclerosis (SSc) is a connective tissue disease characterized by fibrosis of the skin and internal organs due to fibroblast proliferation and excessive production of extracellular matrix (ECM) [1]. The mechanism(s) resulting in fibrosis in SSc are still under investigation. There are currently no effective treatments to prevent or halt the progression of fibrosis in SSc or other fibrosing diseases [2]. SSc has a worldwide distribution and is more frequent in women than men [3]. The female:male ratio is approximately 3:1, but this ratio increases to 10:1 during the child-bearing years [1]. Female sex hormones such as estrogens may thus contribute to disease pathogenesis.

There are three main estrogens: estradiol, estrone, and estriol. Estradiol and estrone are the estrogens found in nonpregnant women, while estriol is the estrogen of pregnancy. Estrogens, especially 17β-estradiol (E2), play an important role in many normal physiological processes in mammals such as reproduction, cardiovascular health, bone integrity, cognition, and behavior [3]. Given this widespread role for E2 in human physiology, E2 is also implicated in the development or progression of numerous diseases - including various types of cancer (breast, ovarian, colorectal, prostatic, endometrial), osteoporosis, neurodegenerative diseases, cardiovascular disease, insulin resistance, endometriosis, and obesity [4-8]. In many of these disorders, estrogen mediates its effects through the estrogen receptors (ERs), which serve as the targets for many therapeutic interventions.

The clinical effects of hormone replacement therapy (HRT) and tamoxifen, a selective ER modulator, have been evaluated in SSc patients [9,10]. HRT was suggested to exert protective effects against the development of isolated pulmonary hypertension in patients with SSc and limited cutaneous involvement [9], while tamoxifen did not improve SSc symptoms [10]. We examined the effects of E2 on fibronectin (FN), an important component of the ECM, and on the development of dermal fibrosis in human skin in organ culture. We also compared estrogen levels in sera of patients with diffuse cutaneous SSc and healthy controls.

## Materials and methods

### Source of fibroblasts

Skin-punch biopsies were obtained with informed consent under an Institutional Review Board-approved protocol at the University of Pittsburgh from the clinically affected and unaffected skin of six patients with SSc and five healthy twins from an existing twin cohort [11,12]. Healthy twins were used as controls since they share the genetic background as the SSc patients. All SSc patients had diffuse skin thickening and met the American College of Rheumatology preliminary criteria for classification as SSc [1].

Biopsies were performed on the leading edge of dermal thickening and clinically normal skin. The skin samples were minced, placed in 60 mm tissue culture dishes, and cultured at 37°C in a humidified atmosphere in DMEM (Cellgro, Herndon, VA, USA) supplemented with 10% fetal bovine serum (Sigma-Aldrich, St Louis, MO, USA), 100 IU/ml penicillin, and 100 μg/ml streptomycin (Invitrogen, Carlsbad, CA, USA).

### Serum samples

Serum was obtained from postmenopausal patients with diffuse cutaneous SSc (*n *= 68) and from age-matched and sex-matched healthy controls (*n *= 35). Both groups had no exposure to HRT. The average age of the SSc patients was 67.6 ± 5.2 years and that of controls 66 ± 0.84 years (not significant). Patients with SSc had disease duration <3 years, with onset defined as the time of the first symptom attributable to SSc.

### Treatment of cells with 17β-estradiol, ER ligands and 17β-estradiol signaling inhibitors

Skin fibroblasts (2×10^5 ^cells per well) were seeded in 35 mm cell culture dishes in DMEM/10% fetal bovine serum. The following day, the medium was replaced with phenol-red free DMEM (Cellgro) without serum for 24 hours to deprive the cells of estrogen. Fresh phenol red-free DMEM plus 10% charcoal-stripped fetal bovine serum (Hyclone, Logan, UT, USA) was added with one of the following: ethanol as vehicle control (0.1%) or E2 (10 nM; Sigma-Aldrich) for 24 hours (for RNA extraction) or 48 hours (for protein extraction). Transforming growth factor beta (10 ng/ml; R&D Systems, Minneapolis, MN, USA) was used as a positive control. ICI 182,780 (100 nM; Tocris, Ballwin, MO, USA), a pure ER antagonist, and signaling inhibitors (MEK inhibitor U0126, inositol-1,4,5-triphosphate (PI3K) inhibitor LY294002, and p38 mitogen-activated protein kinase (MAPK) inhibitor SB202190, 10 μM each; Cell Signaling Technology, Beverly, MA, USA) were added where indicated. To determine the role of ERα and ERβ on FN individually, cells were cultured with propyl-pyrazole-triol (PPT), an ERα specific ligand (100 nM; Tocris) [13], and genistein, an ERβ selective ligand (100 nM; Sigma-Aldrich), under similar conditions to those used for E2 treatment.

### Extracellular matrix extraction

ECM was extracted as we have described previously [14]. Briefly, cells were rinsed with PBS and incubated with 8 M urea in PBS for 20 minutes. Cells were aspirated and the ECM was rinsed three times with PBS. ECM from an equal number of cells was scraped in 100 μl sample buffer (20 mM dithiothreitol, 6% SDS, 0.25 M Tris, pH 6.8, 10% glycerol, 10 mM NaF, and bromophenyl blue) and analyzed by western blot. Equal volumes of ECM were loaded in each lane.

### RNA isolation and RT-PCR

Skin fibroblasts in early passage (passages 3 and 4) were harvested and RNA was extracted using TRIzol (Invitrogen). mRNA was reverse transcribed using Superscript II (Invitrogen) following the manufacturer's recommendations. The cDNA generated was used as a template for amplification by PCR with primers specific for FN, 5'-ACCGTGTGGGTACAGGTG-3' and 5'-GTCACAGAGGCTACTAT-3', and β-actin, 5'-ATGTTTGAGACCTTCAACAC-3' and 5'-CACGTCACACTTCATGATGG-3'. PCR amplification was performed in a 50 μl reaction containing Taq DNA polymerase (Invitrogen), 10× PCR buffer (750 mM Tris-HCl, pH 8.8, 200 mM (NH_4_)_2_SO_4 _and 0.1% Tween 20), 1.5 mM MgSO_4_, and 1 mM of each deoxynucleotide triphosphate in a Peltier Thermal Cycler-200 (MJ Research, Waltham, MA, USA).

Conditions were an initial denaturation at 95°C for 4 minutes, followed by 35 cycles of 94°C for 45 seconds, 55°C for 30 seconds, and 68°C for 2 minutes. Final extension was at 68°C for 5 minutes. Then 20 μl each reaction was electrophoresed on a 1% agarose gel in 1× Tris/acetate/ethylenediamine tetraacetic acid buffer and products were visualized following staining with ethidium bromide. The molecular weights of the PCR products were: FN 513 bp and β-actin 494 bp.

### Protein extraction and western blot

Cells were grown to confluency in 35 mm culture dishes. Cells were rinsed with 1× PBS and scraped in sample buffer (20 mM dithiothreitol, 6% SDS, 0.25 M Tris, pH 6.8, 10% glycerol, 10 mM NaF, and bromophenyl blue). Samples were separated by electrophoresis on 8% SDS-polacrylamide gels and transferred to nitrocellulose membranes. Membranes were blocked with 5% nonfat milk in 1× TBS-Tween 20 (0.2 M Tris, 0.14 M NaCl, 0.1% Tween 20), followed by incubation with mouse monoclonal anti-human EDA-FN antibody, rabbit polyclonal anti-human FN antibody, rabbit polyclonal anti-ERα antibody, rabbit polyclonal anti-ERβ antibody (Santa Cruz Biotechnology, Santa Cruz, CA, USA), mouse monoclonal anti-human vitronectin, mouse monoclonal anti-β-actin (Sigma-Aldrich), or mouse monoclonal anti-GAPDH (Ambion, Austin, TX, USA) in 1× TBS-Tween 20. Membranes were then incubated with horseradish peroxide-conjugated donkey anti-rabbit IgG (Amersham, Piscataway, NJ, USA) or donkey anti-mouse IgG (Amersham). Immunoreactive proteins were detected by chemiluminescence (PerkinElmer Life Sciences, Boston, MA, USA), followed by autoradiography.

### Treatment of human skin *ex vivo*

Human abdominal skin was obtained from cosmetic plastic surgery. All tissues were obtained according to the guidelines of the University of Pittsburgh and under a protocol approved by the Institutional Review Board of the University of Pittsburgh. As described previously [15], subcutaneous fat tissue was removed uniformly and samples composed of complete epidermal and dermal strata were cut into 1.5 cm×1.5 cm sections. Skin was maintained in organ culture in the presence of the indicated factors, E2 (10 nM), ICI 182,780 (100 nM), PPT (100 nM), and genistein (100 nM). Skin was harvested, fixed in 10% formalin, and embedded in paraffin.

### Measurement of skin dermal and collagen bundle thickness

Dermal and collagen bundle thickness were measured in skin sections stained with H & E. Dermal thickness was defined as the distance from the granular layer to the junction between the dermis and subcutaneous fat. Images were taken on a Nikon Eclipse 800 microscope (Nikon Instruments, Inc., Huntley, IL, USA) using identical camera settings, and ImageJ (National Institutes of Health, Bethesda, MD, USA) was used to measure thickness. Thickness was measured in five random fields in each sample.

### Immunohistochemistry

Sections (6 μm) of paraffin-embedded skin tissues were de-paraffinized, endogenous peroxidase was quenched using 10% H_2_O_2_, and endogenous biotin was blocked using the biotin blocking kit (Dakocytomation, Carpinteria, CA, USA). The sections were blocked with 5% serum and incubated with anti-FN antibody followed by secondary antibody. Bound secondary antibody was detected using the aminoethyl carbazole Red kit (Invitrogen, Carlsbad, CA, USA). A light hematoxylin counterstain was used to identify nuclei. Images were taken on a Nikon Eclipse 800 microscope.

### Measurement of 17β-estradiol and estrone in serum

Serum levels of E2 and estrone were measured using liquid chromatography-tandem mass spectrometry in the Small Biomolecule Core Facility in the School of Pharmacy at the University of Pittsburgh. The liquid chromatography-tandem mass spectrometry method employs liquid-liquid extraction, derivatization, and detection with a triple quad mass spectrometer using 0.5 ml serum.

### Statistical analysis

For the *in vitro *and *ex vivo *data, statistical comparisons were performed using the Mann-Whitney U test. For the comparison of serum levels of E2 and estrone, two separate sets of analyses were performed: case versus control comparisons of estrone and E2; and case-only comparisons of clinical manifestations based on high, intermediate, and low estrone or E2. For these comparisons, the Wilcoxon rank-sum test, the chi-square test of proportions, and Fisher's exact test were used where appropriate.

## Results

### Effect of 17β-estradiol on fibronectin mRNA and protein levels

**Figure 1:**
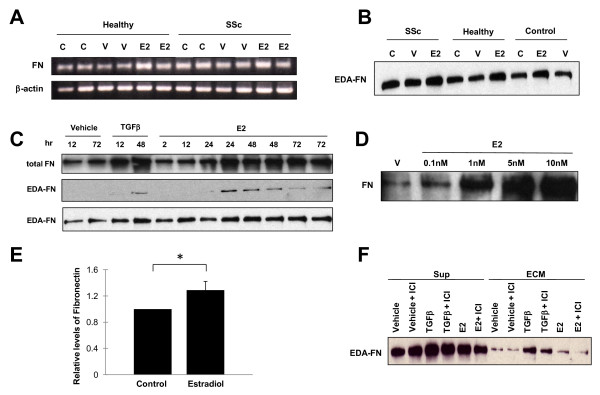
**Estradiol increases fibronectin production in *vitro*. (A) **Fibronectin (FN) mRNA expression in primary skin fibroblasts from twins discordant for systemic sclerosis (SSc). FN mRNA was analyzed after 24-hour treatment with 10 nM 17β-estradiol (E2), vehicle (V), or no treatment (control, C) using RT-PCR. Assays were done in duplicate. **(B) **FN protein expression in culture supernatants of primary skin fibroblasts. FN protein expression was analyzed by western blot in untreated, E2-treated or vehicle-treated fibroblasts for 48 hours. SSc, SSc patient; healthy, healthy twin of an SSc patient; control, unrelated healthy donor. **(C) **FN protein expression in the extracellular matrix (ECM) and culture supernatants (Sup) of normal skin fibroblasts treated with vehicle, transforming growth factor beta (TGFβ), or E2. ECM and culture supernatants were harvested at the indicated time points. **(D) **FN levels in the ECM and Sup of normal skin fibroblasts stimulated with vehicle and different concentrations of E2 ranging from 0.1 to 10 nM. **(E) **Graphical summary of FN levels representing data from four independent experiments. *P = 0.0036. **(F) **Effect of E2 blockade on the expression of FN protein in the ECM and Sup of normal skin fibroblasts stimulated with E2. Cells were treated with E2 for 48 hours in the presence or absence of the ER antagonist ICI 182,780.

The effect of E2 on FN expression was examined using RT-PCR and western blot analysis. In untreated samples, FN mRNA and protein levels in SSc patient fibroblasts were higher than those in their healthy twins. E2 increased FN mRNA and protein levels in healthy twin and SSc fibroblasts (Figure 1A,B). E2 increased FN mRNA and protein levels in a time-dependent and dose-dependent manner in cell supernatants and ECM (Figure 1C,D). E2 induced production of total FN and EDA-domain-containing matrix FN (Figure 1C) and the increase in secreted FN was significant (Figure 1E). The ER antagonist ICI 182,780 blocked the effect of E2 on FN mRNA and protein expression but did not affect transforming growth factor beta-induced FN levels (Figure 1F).

### Signaling pathways mediating the effects of 17β-estradiol on fibronectin induction

**Figure 2:**
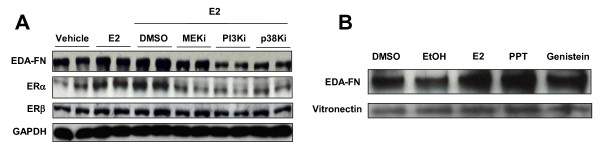
**Estradiol promotes fibronectin production via specific signaling cascades**. **(A) **Fibronectin (FN) protein levels in the cellular lysates of normal skin fibroblasts. Normal skin fibroblasts were stimulated with 17β-estradiol (E2) for 48 hours in the presence or absence of the following chemical inhibitors: MEK inhibitor (MEKi), phosphoinositol 3-kinase inhibitor (PI3Ki), and p38 kinase inhibitor (p38Ki). Cellular lysates were analyzed by western blot using anti-EDA-FN, estrogen receptor (ER)α, ERβ, and GAPDH antibodies. **(B) **Effect of E2 ligands on the expression and deposition of FN in the extracellular matrix (ECM) of normal skin fibroblasts. PPT, propyl-pyrazole-triol. Primary fibroblasts were cultured with vehicle (dimethylsulfoxide (DMSO), ethanol (EtOH)), E2, PPT, or genistein for 48 hours. ECM was analyzed by western blot using anti-EDA-FN and anti-vitronectin antibodies. Ethanol was used as vehicle for E2 and PPT. DMSO was used as a vehicle for genistein.

To investigate the mechanism mediating E2 induction of FN, we pretreated skin fibroblasts with vehicle, MEK inhibitor, PI3K inhibitor, or p38 MAPK inhibitor for 1 hour prior to the addition of E2. FN protein levels were assessed by western blot analysis 48 hours post treatment. PI3K inhibitor and p38 MAPK inhibitor attenuated the E2-mediated increase of FN (Figure 2A). MEK inhibitor had a more modest effect on E2 induction of FN. We also examined the effect of the chemical inhibitors on ERα and ERβ. ERα was increased by E2 and this increase was blocked by PI3K inhibitor, p38 MAPK inhibitor, and MEK inhibitor. There was no significant difference in the expression of ERβ under the same conditions (Figure 2A).

### Effect of ER ligands on fibronectin expression

To assess the individual effects of ERα and/or ERβ on FN expression, we used PPT, an ERα ligand, and genistein, an ERβ ligand. Primary fibroblasts were treated with vehicle, E2, PPT, or genistein for 48 hours. ECM was harvested and analyzed by western blot. Vitronectin was detected as an ECM loading control. E2 and PPT increased FN protein levels in the ECM (Figure 2B). Genistein modestly increased FN protein levels (Figure 2B). Vitronectin levels were not altered by any of the treatments.

### Estradiol and ERα agonist induce increased dermal and collagen bundle thickening and fibronectin deposition in human skin

**Figure 3:**
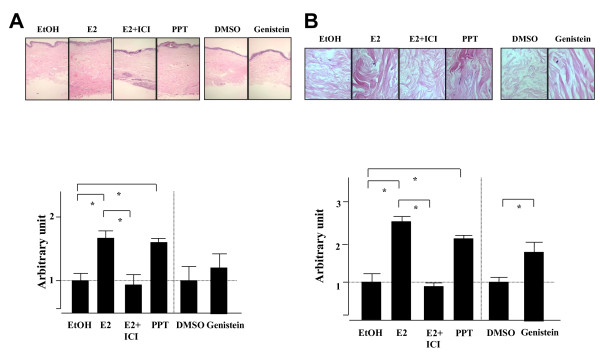
**Estradiol promotes the development of fibrosis *ex vivo *in human skin**. **(A) **Estrogen and estrogen receptor (ERα) agonist induce skin thickening *ex vivo*. Human skin samples were plated on six-well plates and treated with ethanol (EtOH), 17β-estradiol (E2), estradiol with ICI 182,780 (E2+ICI), propyl-pyrazole-triol (PPT), dimethylsulfoxide (DMSO), and genistein for 7 days. **(A) **Images of H & E-stained sections taken at 40× magnification. Skin thickness was measured and the ratio of the thickness compared with vehicle control (EtOH or DMSO) was calculated as an arbitrary unit. Data shown are from three independent experiments. *P >0.05 by Mann-Whitney U test. **(B) **Estrogen and ERα agonist also induce collagen bundle thickening in *ex vivo*. Human skin samples were treated with EtOH, E2, E2+ICI, PPT, DMSO, or genistein for 7 days. Images of H & E-stained sections are shown. Images taken at 800× magnification. Thickness of collagen bundles was measured and the ratio compared with vehicle control (EtOH or DMSO) was calculated as an arbitrary unit. Data shown are from three independent experiments. *P >0.05 by Mann-Whitney U test.

**Figure 4:**
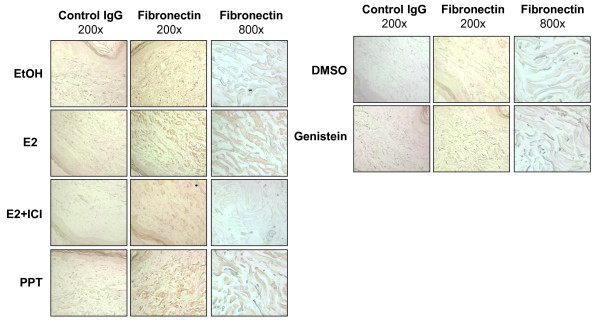
**Estradiol induces fibronectin production *ex vivo *in human skin**. Estrogen and estrogen receptor alpha (ERα) agonist induce fibronectin expression *ex vivo*. Human skin samples were plated on six-well plates and treated with ethanol (EtOH), 17β-estradiol (E2), estradiol with ICI 182,780 (E2+ICI), propyl-pyrazole-triol (PPT), dimethylsulfoxide (DMSO), and genistein and incubated for 7 days. Immunohistochemistry was performed using anti-fibronectin antibody and visualized with aminoethyl carbazole (red).

To further examine the effect of E2 in skin tissues, the dermal and collagen bundle thicknesses in dermis were assessed using an *ex vivo *organ culture system. Explanted skin tissues on 35 mm well plates were treated with E2, ERα or ERβ agonists (PPT or genistein, respectively), or vehicle (ethanol for E2 and PPT, and dimethylsulfoxide for genistein) for 7 days, and skin sections were stained with H & E. As shown in Figure 3, E2 and PPT induce increase of dermal thickness (Figure 3A) and collagen bundle thickness (Figure 3B) compared with vehicle (dermis: 1.61 ± 0.12, *P *<0.05 and 1.54 ± 0.05, *P *<0.05, respectively; collagen bundles: 2.62 ± 0.18, *P *<0.05 and 1.84 ± 0.15, *P *<0.05, respectively), and ICI 182,780 blocked the effect of E2. On the contrary, genistein did not induce thickening of dermis or collagen bundles (Figure 4A,B). We also assessed the extent of deposition of FN using immunohistochemistry. As shown in Figure 4, the results of FN deposition in collagen bundles were similar to those for thickness of skin and collagen bundles. E2 thus induces skin fibrosis, and this effect is mediated by ERα.

### Circulating levels of 17β-estradiol and estrone are significantly increased in postmenopausal patients with systemic sclerosis

**Figure 5:**
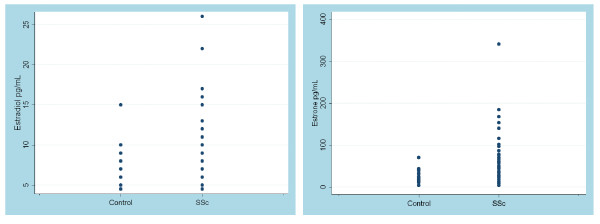
**Estradiol and estrone levels are increased in serum of patients with diffuse cutaneous systemic sclerosis**. Dot plots of serum levels of estradiol and estrone in patients with diffuse cutaneous systemic sclerosis (SSc) and healthy controls. Measurements were made using 68 patients with SSc and 35 healthy controls. Differences in estradiol levels were not significant whereas estrone levels were statistically significant (*P *= 0.002).

Patient and control E2 serum samples were divided into low (<5 pg/ml), intermediate (5 to 10 pg/ml), and high (>10 pg/ml) levels (Table 1). Similarly, patient and control estrone serum samples were divided into low (<15 pg/ml), intermediate (15 to 75 pg/ml), and high (>75 pg/ml) levels (Table 1). There was a significant difference between SSc patient and control E2 and estrone levels (*P *= 0.04 and *P *= 0.006, respectively). The frequency of the data points is shown in the dot plots of Figure 5. Levels of E2 and estrone were also analyzed by disease specific clinical manifestations occurring at any time during the illness. Although the associations did not reach statistical significance, a larger proportion of patients with high estrone levels (42%) had gastrointestinal involvement compared with those patients with low estrone levels (17%, *P *= 0.07).

**Table 1:** Levels of estradiol and estrone in patients with diffuse cutaneous systemic sclerosis and healthy controls

	Low	Intermediate	High	Total
Estradiol^a^				
Controls	14	20	1	35
dcSSc	34	24	10	68
Total	48	44	11	103
Estrone^b^				
Controls	7	18	0	25
dcSSc	9	31	15	55
Total	16	49	15	80

^a^Low (<5 pg/ml), intermediate (5 to 10 pg/ml), and high (>10 pg/ml); *P *= 0.04 for controls versus diffuse cutaneous systemic sclerosis (dcSSc) using chi-square/Fisher's exact test. ^b^Low (<15 pg/ml), intermediate (15 to 75 pg/ml), and high (>75 pg/ml); *P *= 0.006 for controls versus dcSSc using chi-square/Fisher's exact test.

## Discussion

We present data establishing a role for E2 in the induction of a fibrotic phenotype. E2 was previously demonstrated to increase collagen during wound healing [16,17]. We and others have previously reported that FN mRNA levels in SSc dermal fibroblasts are up to 10-fold greater than those in healthy donors [18,19]. E2 increases FN mRNA in cardiac fibroblasts and this increase was associated with ECM remodeling [20]. However, the mechanism(s) mediating the effect of E2 on FN expression are poorly understood. To our knowledge, this report is the first to delineate the mechanisms mediating E2 induction of FN in human skin.

SSc is more frequent in women than men and the female:male ratio further increases to 10:1 during the child-bearing years [1,3]. E2 levels in women during the child-bearing years are significantly higher than those in postmenopausal women. The menstrual cycle has four phases (menstrual, follicular, ovulatory, and luteal), and each phase is characterized by different circulating levels of E2 [21]. These E2 levels during ovulation are 490 to 1,710 pmol/l (mean 1,087 pmol/l) and exceed levels detected during the other phases [22]. E2 levels in postmenopausal women are 2 to 18 pg/ml (mean 7.6 pg/ml) [23], which is equivalent to 28 pmol/l and significantly lower than levels in women of child-bearing age. Circulating E2 levels are thus increased in the age range during which the SSc female:male ratio is highest. E2 levels that promoted a fibrotic phenotype in our assays were physiological and ranged from 0.1 to 10 nM. These levels were similar to levels measured in the circulation of women during ovulation (average 1 nM) [22].

Our data confirm the expression of ERα and ERβ in primary dermal fibroblasts [24]. We further show that PPT, an ERα specific ligand, increases FN production. Moreover, ERα is increased by E2 treatment of skin fibroblasts. These results suggest that ERα is the main regulator of E2-mediated FN expression in dermal fibroblasts. Interestingly, ERβ levels were much lower in SSc patient fibroblasts than in healthy twin fibroblasts (data not shown). ERβ expression is decreased in colon and prostate cancers and its reduced expression is related to tumor cell dedifferentiation [25-30]. Global antagonism of ERα transcriptional activity by ERβ has been reported [31]. ERβ represses several ERα-mediated effects, including fat reduction and cellular proliferation in the uterus and prostate [31]. We further show that E2, acting via ERα, exerts profibrotic effects. The FN-promoting effects of E2 were confirmed *in vitro *in dermal fibroblasts during the preparation of this manuscript by Soldano and colleagues [32]. These effects are probably tissue specific, however, since E2 attenuates tubulointerstitial fibrosis in diabetic nephropathy [33]. In summary, our findings suggest that ERβ could play a protective role in SSc. A similar antifibrotic role for ERβ was recently reported in a model of cardiac fibrosis [34]. Further studies are needed to determine whether ERα and ERβ can exert converter-regulatory effects in the modulation of FN expression in SSc and normal dermal fibroblasts.

ER acts as a ligand-activated transcription factor. The classical mechanism of ER action involves estrogen binding to nuclear receptors followed by receptor dimerization and binding to specific response elements known as estrogen response elements located in the promoters of target genes. Dimerized receptors can also bind other transcription factors such as AP-1 and SP-1 [35-37]. Estrogens exert some of their effects through the action of ERs on gene expression, but a number of other effects of estrogens are so rapid that they cannot rely on the activation of RNA or protein synthesis. These actions are known as nongenomic actions and are believed to be mediated through membrane-associated ERs. Most endogenous plasma membrane ERs exist as homodimers in the presence of E2 [38] and mediate rapid E2 activation of a number of signaling cascades, including cyclic AMP, PI3K, phospholipase C, and MAPK [39]. These signaling pathways regulate cytokine production, apoptosis, cell cycle arrest, regulation of RNA splicing or stabilization, and tumor cell differentiation [40,41].

The MAPK superfamily consists of three well-characterized subfamilies [42]. Extracellular signal-regulated kinases respond to growth factors or other external mitogenic signals and are involved in promoting cell proliferation. The p38 MAPK and c-Jun N-terminal kinase pathways are distinguished by generally being activated in response to stress and are thus called the stress-activated kinases that promote inflammation and programmed cell death [43]. PI3K also has an important role in mitosis, apoptosis, motility, proliferation, and differentiation [44]. We have demonstrated that all three kinases (extracellular signal-regulated kinase MAPK, p38K MAPK and PI3K) regulate E2 signaling and its induction of FN expression, with FN induction being mainly regulated by PI3K and p38 MAPK and to a lesser extent by extracellular signal-regulated kinase MAPK. PI3K and p38 MAPKs have also been reported to regulate E2/ER's anti-apoptotic action on cardiomyocytes [41]. Our findings support the role of these E2 signaling cascades in skin fibroblasts and in the regulation of ECM production.

We had previously shown that human skin maintained in an organ culture system can be used to recapitulate *in vivo *events and to test the efficacy of antifibrotic agents [13]. Our current data demonstrate that E2 can exert profibrotic activity *ex vivo *in human skin and that this effect can be specifically blocked by ICI 182,780. The extension of our data describing the profibrotic effects of E2 to human tissues supports the applicability of our findings to human disease and the potential therapeutic effects of ICI 182,780 for human fibrosis.

The preponderance of SSc in women suggests that estrogens play a role in disease pathogenesis. We show that circulating E2 and estrone levels are elevated in postmenopausal patients with diffuse cutaneous SSc compared with healthy women, implicating estrogens, and specifically E2 and estrone, in the disease process. Numerous studies have shown that dermal skin thickness and collagen content increase in women on estrogen replacement therapy [45,46]. Furthermore, clinical trials have shown that postmenopausal women on HRT have thicker skin compared with women not taking HRT [47-50]. The profibrotic role of E2 has been confirmed in the bleomycin-induced rat lung fibrosis model where female animals had a more profound fibrotic response compared with males, which was attenuated following ovariectomy and accentuated with HRT [51]. In mice, castration decreases skin thickness and ovariectomy reduces expression of matrix-associated proteoglycans [52], suggesting that the absence of sex steroid hormones reduces expression of ECM components. These reports further support the role of estrogens in the development of fibrosis in SSc and suggest that E2 can be a trigger of ECM production and fibrosis.

Estrogen has been implicated in autoimmune diseases based on its ability to promote B-lymphocyte survival and activation, thus facilitating autoreactivity [53]. In the setting of inflammation, accelerated conversion of androgens to estrogen metabolites via aromatase occurs in the peripheral tissues [53]. This peripheral conversion may contribute to increased E2 levels in postmenopausal patients with SSc. Concentrations of E2 in skin from individuals with SSc probably exceed those detected in the circulation due to local hormone production mediated by aromatase [53]. Our *ex vivo *human skin model mimics the effect of peripheral estrogens found in postmenopausal women with SSc. In autoimmunity, conversion is accelerated by the induction of aromatase activity by inflammatory cytokines such as IL-6, which is increased in autoimmune diseases including SSc [54,55].

## Conclusion

We have identified E2 as an inducer of FN expression in skin fibroblasts obtained from SSc patients and healthy donors. The effects of E2 on FN were mainly regulated via ERα and the E2/ER downstream signaling cascades, PI3K and p38 MAPK. We also demonstrated that E2 is fibrotic *ex vivo *and that ICI 182,780 can be used effectively to inhibit dermal fibrosis. The profibrotic effect of E2 and the increased circulating levels of E2 and estrone may explain, at least in part, the higher frequency of SSc in women.

## Abbreviations

bp: base pairs; DMEM: Dulbecco's modified Eagle's medium; E2: 17β-estradiol; ECM: extracellular matrix; ER: estrogen receptor; FN: fibronectin; H & E: hematoxylin and eosin; HRT: hormone replacement therapy; IL: interleukin; MAPK: mitogen-activated protein kinase; PBS: phosphate-buffered saline; PCR: polymerase chain reaction; PI3K: inositol-1,4,5-triphosphate; PPT: propyl-pyrazole-triol; RT: reverse transcriptase; SSc: systemic sclerosis.

## Competing interests

The authors declare that they have no competing interests.

## Authors' contributions

KA-Y, CP, HY, KT, PH, and CAF-B contributed to the *in vitro *assays. CP, JAC, TAM, and CAF-B contributed to the serum analysis studies. KA-Y, HY, and CAF-B contributed to the *ex vivo *human skin studies. All authors contributed to drafting and editing of the manuscript. All authors read and approved the manuscript for publication.
